# Cost-utility of screening with liquid cytology or p16/Ki67 dual stain in women identified in cervical cancer triage with non 16/18 HR-HPV

**DOI:** 10.1017/S0266462326103675

**Published:** 2026-03-30

**Authors:** Isandra Meirelles, Marcia Pinto, Fabio Russomano

**Affiliations:** Fundação Oswaldo Cruz - Instituto Fernandes Figueira, Rio de Janeiro, Brazil

**Keywords:** cost-effectiveness analysis, uterine cervical neoplasms, dual stain, HPV, triage, cervical cancer screening

## Abstract

**Objectives:**

To perform a cost-utility analysis of the p16/Ki67 dual stain compared to liquid cytology (LC) in the screening of women aged 25–60 years with high-risk papillomavirus (HR-HPV) non 16/18, from the perspective of the Sistema Único de Saúde (SUS) of Brazil.

**Methods:**

A Markov-coupled decision tree cost-utility analysis model was developed for the follow-up of a hypothetical cohort of 1,000 women in the age group, in health states that simulated the natural progression of cervical cancer. The time horizon was lifetime with a discount rate of 5 percent for costs and benefits. To survey the resources used for the procedures involved, the recommendations of the Brazilian Guidelines for Cervical Cancer Screening were considered. Most of the cost data were obtained from SUS administrative and public databases. Deterministic and probabilistic sensitivity analyses were carried out.

**Results:**

Screening with p16/Ki67 outperformed LC, resulting in an incremental gain of 2.5 quality-adjusted life years (QALYs) and an incremental cost-effectiveness ratio of R$31.40/QALY (range R$12.98–62.90/QALY), well below the reference value of R$40,000/QALY.

**Conclusions:**

The p16/Ki67 test proved to be cost-effective in screening women with non 16/18 HR-HPV. The results can help Brazilian managers plan and make decisions about incorporating technologies for triage considering the use of HPV tests in cervical cancer screening.

## Introduction

Molecular testing for the detection of human papillomavirus (HPV) is currently recognized as the recommended strategy for cervical cancer screening, replacing cytology in several countries, replacing cytology in several countries. In 2024, HPV testing was incorporated into the Sistema Único de Saúde (SUS), and Brazil is currently in the transition phase for this new method, with the screening guideline being updated.

One of the main challenges for the implementation of primary HPV screening using molecular tests is the risk of excessive referrals for colposcopy and overtreatment, due to the lower specificity of the test, which reinforces the need for an effective and efficient screening strategy (1;2).

In the Brazilian context, the Comissão Nacional de Tecnologias em Saúde (Conitec) recommends the use of partial or extended genotyping. For high-risk HPV (HR-HPV) genotypes other than 16 or 18, reflex liquid cytology (LC) is indicated to triage positive cases and reduce unnecessary referrals. In contrast, women testing positive for HPV 16/18 should be referred directly to colposcopy, given the higher oncogenic potential and stronger association of these genotypes with cervical cancer (3;4). Nevertheless, the limited sensitivity of cytology reduces its negative predictive value, which increases the likelihood of false-negative results (5).

The prospective IMPACT trial, involving over 35,000 women in the United States, showed that p16/Ki-67 dual stain (DS) achieved a sensitivity of 83 percent and specificity of 57 percent for non 16/18 HR-HPV, compared to 59 percent sensitivity and 66 percent specificity for cytology (6). In 2024, the World Health Organization (WHO) reinforced the recommendation of preferential use of DS over reflex cytology in these cases (7).

Despite this evidence, the best screening strategy for women with non 16/18 HR-HPV in Brazil has yet to be defined from an economic point of view. Therefore, this study aims to carry out a cost-utility analysis to assess which of these technologies offers good value for money for screening these women from the perspective of the SUS.

## Methods

### Model structure and main parameters

**Figure 1.**
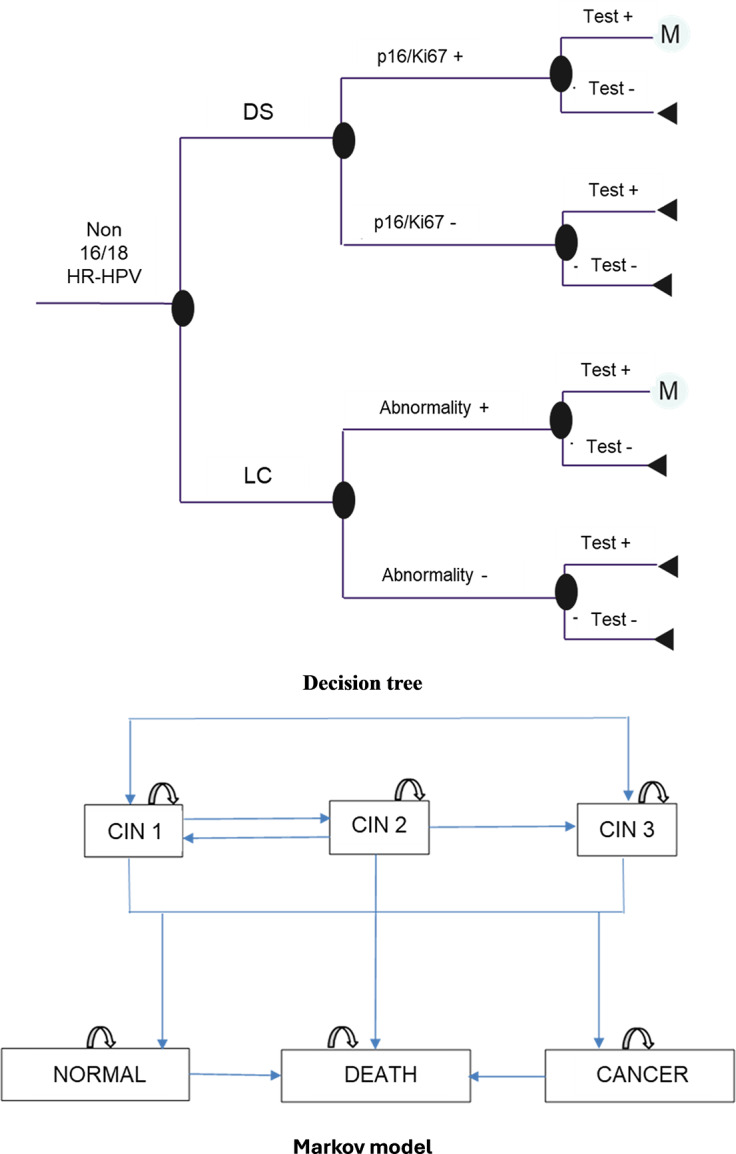
Decision model.

A decision tree model combined with a Markov chain (Figure 1) was developed using Microsoft 365 Excel (Microsoft Corporation, Redmond, WA, USA). Annual cycles were defined based on the greatest availability of published data on disease progression, primarily derived from a recent high‑quality meta-analysis (8). The probabilities of regression or progression of cervical intraepithelial neoplasia (CIN) were predominantly reported over a 12‑month period. This timeframe aligns with the evaluation period of tests’ performance in the IMPACT clinical trial (6).

The parameters were filled in preferably with data from scientific literature or local databases, when available, and their estimates and limits for sensitivity analysis are described at Table 1.

**Table 1.** Summary of parameters and values varied in the deterministic sensitivity analysis

Parameter description	Point estimate	Lower limit	Upper limit	Reference
Probability of transition – CIN 1 to CIN 1*	0.25	–	–	Loopik et al. (8)
Probability of transition – CIN 1 to CIN 2*	0.10	–	–	Loopik et al. (8)
Probability of transition – CIN 1 to CIN 3*	0.01	–	–	Loopik et al. (8)
Probability of transition – CIN 1 to Normal*	0.64	–	–	Loopik et al. (8)
Probability of transition – CIN 1 to Cancer*	0.0002	–	–	Loopik et al. (8)
Probability of transition – CIN 1 to Death*	0.0002	–	–	Assuming the same mortality rate as the general population
Probability of transition – CIN 2 to CIN 1*	0.31	–	–	Loopik et al. (8)
Probability of transition – CIN 2 to CIN 2*	0.12	–	–	Loopik et al. (8)
Probability of transition – CIN 2 to CIN 3*	0.10	–	–	Loopik et al. (8)
Probability of transition – CIN 2 to Normal*	0.46	–	–	Loopik et al. (8)
Probability of transition – CIN 2 to Cancer*	0.0015	–	–	Loopik et al. (8)
Probability of transition – CIN 2 to death*	0.0002	–	–	
Probability of transition – CIN 3 to CIN 1*	0.28	–	–	Loopik et al. (8)
Probability of transition – IAS 3 to IAS 3*	0.67	–	–	Loopik et al. (8)
Probability of transition – CIN 3 to Normal*	0.04	–	–	Loopik et al. (8)
Probability of transition – CIN 3 to Cancer*	0.0101	–	–	Loopik et al. (8)
Probability of transition – CIN 3 to Death*	0.0002	–	–	Assuming the same mortality rate as the general population
Probability of transition – Cancer to Cancer*	0.92	–	–	Loopik et al. (8)
Probability of transition – Cancer to Death*	0.08	–	–	Assuming the same mortality rate as the general population
Probability of transition – Normal to Normal*	0.9998	–	–	Completion of the probability of death
Probability of transition – Normal to Death*	0.0002	–	–	Assuming the same mortality rate as the general population
Average entry age – 25–34 group	30	25	34	Average age between the two limits
Average entry age – 35–60 group	48	35	60	Average age between the two limits
Sensitivity of liquid cytology (CIN 2+)	59%	53.2%	64.2%	Wright (6)
Specificity of liquid cytology (CIN 2+)	65.5%	63.6%	67.4%	Wright (6)
p16/Ki67 sensitivity (CIN 2+)	83%	78.4%	86.8%	Wright (6)
Specificity of p16/Ki67 (CIN 2+)	57%	54.8%	58.8%	Wright (6)
Frequency of p16/Ki67+	28%	11.50%	35.20%	Trzeszcz (34); Possati-Resende (35)
Frequency of abnormalities in cytology	3.9%	2.75%	8.80%	Teixeira (36); Kawamura (5); dos Santos (37)
Prevalence of CIN^1^ 1 – 25–29 years old	41.5%	15%	68%	SISCAN^3^ (10)
Prevalence of CIN 1 – 30–34 years old	40.7%	15%	69%	SISCAN (10)
Prevalence of CIN 1 – 35–39 years old	39.0%	16%	69%	SISCAN (10)
Prevalence of CIN 1 – 40–44 years old	38.5%	12%	77%	SISCAN (10)
Prevalence of CIN 1 – 45–49 years old	30.8%	12%	62%	SISCAN (10)
Prevalence of CIN 1 – 50–54 years old	25.5%	11%	53%	SISCAN (10)
Prevalence of CIN 1 – 55–60 years old	17.5%	6%	42%	SISCAN (10)
Prevalence of CIN 2 – 25–29 years old	15.7%	7%	25%	SISCAN (10)
Prevalence of CIN 2 – 30–34 years old	13.1%	7%	20%	SISCAN (10)
Prevalence of CIN 2 – 35–39 years old	12.5%	5%	23%	SISCAN (10)
Prevalence of CIN 2 – 40–44 years old	9.1%	4%	17%	SISCAN (10)
Prevalence of CIN 2 – 45–49 years old	8.0%	3%	16%	SISCAN (10)
Prevalence of CIN 2 – 50–54 years old	4.5%	2%	10%	SISCAN (10)
Prevalence of CIN 2 – 55–60 years old	4.5%	1%	11%	SISCAN (10)
Prevalence of CIN 3 – 25–29 years old	12.2%	3.8%	20.6%	SISCAN (10)
Prevalence of CIN 3 – 30–34 years old	12.2%	5.4%	19.7%	SISCAN (10)
Prevalence of CIN 3 – 35–39 years old	14.5%	4.7%	27.2%	SISCAN (10)
Prevalence of CIN 3 – 40–44 years old	10.6%	3.4%	21.1%	SISCAN (10)
Prevalence of CIN 3 – 45–49 years old	5.9%	2.6%	11.3%	SISCAN (10)
Prevalence of CIN 3 – 50–54 years old	3.8%	2.6%	6.1%	SISCAN (10)
Prevalence of CIN 3 – 55–60 years old	2.8%	0.6%	7.7%	SISCAN (10)
Prevalence of cervical cancer – 25–29 years old	0.3%	0.1%	0.5%	SISCAN (10)
Prevalence of cervical cancer – 30–34 years old	0.48%	0.45%	0.51%	SISCAN (10)
Prevalence of cervical cancer – 35–39 years old	0.9%	0.2%	1.8%	SISCAN (10)
Prevalence of cervical cancer – 40–44 years old	0.6%	0.3%	1.0%	SISCAN (10)
Prevalence of cervical cancer – 45–49 years old	0.7%	0.3%	1.2%	SISCAN (10)
Prevalence of cervical cancer – 50–54 years old	0.15%	0.1%	0.20%	SISCAN (10)
Prevalence of cervical cancer – 55–60 years old	1.8%	0.6%	4.5%	SISCAN (10)
Cost of liquid cytology	R$13.84	R$13.72	R$25.22	SIGTAP4 (25); SIA (22); Laboratório Central de Tocantins
Cost of the p16/Ki67 test^7^	R$196.21	R$194.24	R$357.09	Central Laboratory of Tocantins
Average cost of colposcopy	R$4.28	R$3.46	R$7.45	SIA5, April/2023-April/2024 (15)
Average cost of biopsy	R$21.84	R$19.04	R$28.75	SIA, April/2023-April/2024 (15)
Average cost of excisional procedures	R$226.30	R$197.22	R$257.68	SIA, 3April/2023-April/2024 (15) and SIH6 April/2023-April/2024 (16)
Cost of cancer treatment	R$35,477.90	R$18,697.25	R$40,444.81	Recommendation Report No. 878 (3); Santos (38)
Utility of the cancer state	0.60	0.41	0.69	Conway (39) adjusted by the Brazilian utility table (27)
Disutility of CIN 1 status	0.002	–	–	Drolet (9)
Disutility of CIN 2 status	0.003	–	–	Drolet (9)
Disutility of CIN 3 status	0.003	–	–	Drolet (9)
Annual discount rate – costs	5%	0%	10%	Methodological Guidelines: Health Economic Evaluations (28)
Annual discount rate – effectiveness	5%	0%	10%	Methodological Guidelines: Health Economic Evaluations (28)

*Note:* *Parameters without upper and lower limits were not varied in the deterministic sensitivity analysis. 1. RCT = randomized clinical trial; 2. CIN = cervical intraepithelial neoplasia; 3. SISCAN = Cancer Information System; 4. SIGTAP = Information System of the SUS Table of Procedures, Medicines and OPM; 5. SIA = Outpatient Information System; 6. SIH = Hospital Information System; 7. Arbitrary variation following the percentage of variation found for liquid cytology.

A hypothetical cohort of 1,000 women aged 25–60 years with a primary screening result of non 16/18 HR-HPV entered in the model through the decision tree. The age range of participants was defined according to the recommendations for HPV screening in the Brazilian Cervical Cancer Screening Guidelines (9).

Women with CIN or cancer detected after a positive test (LC or p16/Ki67 DS) and confirmed by colposcopy, continued into the Markov, and were followed up until the end of their lives (average life expectancy of 79 yr).

The model considered the natural history of the disease, with health states including CIN 1–3, cervical cancer, normal (healed lesions), and death, allowing for regression, stability, or progression of lesions over the time horizon.

The probabilities of a positive result in both tests were associated with their respective sensitivity and specificity (Table 1), taken from the IMPACT clinical trial (6), the best available comparative evidence of the performance of the two tests in the target population.

The true-positive cases were followed by the Markov model, with entry into the health states determined by the prevalence rates, stratified by age group, of CIN 1, 2, 3 and cancer, extracted from the administrative database of the Cancer Information System with histopathological data from 2023 of women with atypical squamous cells of undetermined significance (ASC-US) or low-grade intraepithelial lesions (LSIL) on primary cytology (10).

False-positive cases were not included in the Markov model on the assumption that colposcopy would identify the absence of a treatable lesion. It was assumed that these women would not return for a new round of screening.

The transition probabilities (Table 1) were taken from the meta-analysis of the natural history of CIN by Loopik et al. (8). Considering the low probability of progression to cancer after treatment of CIN 2/3 (8;11), we opted for a single cancer state in the model, regardless of stage, for simplification purposes.

The probabilities of death in the CIN and Normal states were considered the same as those of the general population, based on the life table of the Instituto Brasileiro de Geografia e Estatística (IBGE) (12), while the transition from Cancer to Death was estimated based on a meta-analysis, conducted by the authors, of Brazilian observational studies (13–19). In accordance with the Methodological Guidelines for Economic Evaluation in Health in Brazil, a discount rate of 5 percent per year was adopted for costs and for the *quality-adjusted life years (QALY)* (20).

### Cost analysis

As the analysis was conducted from the perspective of the SUS, only direct costs were included (21), estimated in 2024 Reais. Most of the costs were taken from the Ambulatorial Information System (22) and Hospital Information System (23), covering both federal transfers and state and municipal contributions between April 2023 and April 2024 (Table 1).

The cost of the DS test (Table 1) was obtained from a bid from the Tocantins Central Laboratory for the purchase of the kit and supplies used to analyze samples from a cross-sectional study of women diagnosed with ASC-US or LSIL (24).

The cost of LC (Table 1) followed the value established in the SUS Procedures and Medication Table Management System (25).

The resources used in the CIN states and their respective frequencies were based on the procedures recommended by the Brazilian Cervical Cancer Screening Guidelines (26).

The cost associated with the Cancer state (Table 1) was estimated based on the treatment values per stage, according to the Conitec report (3) evaluating molecular testing for HPV detection in cervical cancer screening, weighted by the frequencies of diagnosis per stage of the disease (17).

### Health outcomes

The primary outcome of this analysis was the QALY. The utility of the general Brazilian population (27) was assigned to women with a negative screening test (decision tree model) and to those in the Normal state of the Markov model (Table 1).

For the CIN 1, CIN 2, and CIN 3 states, utilities extracted from the literature were applied (Table 1), measured using the EQ-5D instrument in women informed about these cytological alterations (9), which were adjusted for age and applied proportionally to the length of time spent in each state. The utility of the Cancer state (Table 1) was taken from the study by Conway et al. (24), with adjustments for the Brazilian population (27).

### Sensitivity analysis

In the deterministic sensitivity analysis, the lower and upper limits of the prevalence and cost parameters (Table 1) were based on the minimum and maximum values identified in the databases consulted. For parameters from the literature, the confidence interval reported in the respective studies was used (Table 1). In the absence of specific estimates of variation, the percentage variation in the cost of cytology was used as a *proxy* (Table 1).

The probabilistic sensitivity analysis followed statistical distributions appropriate to the type of parameter considered. Specifically, beta distributions were assigned to parameters representing probabilities and utilities, such as sensitivity, specificity, probability of p16/Ki67 positivity, and cytological abnormality detection. Uniform distributions were used for age and discount rate, gamma distributions for cost parameters, and Dirichlet distributions for the prevalence of CIN and cervical cancer.

These choices were based on standard recommendations for health economic modeling, as described by Briggs et al. (28). The complete list of analyzed parameters and assigned distributions can be found in Supplementary Table S1.

## Results

Screening with p16/Ki67 outperformed LC, resulting in an incremental gain of 2.5 QALYs. The incremental cost-effectiveness ratio (ICER) was estimated at R$49.76 per additional QALY (Table 2). Detailed cost and quality of life results by health status are reported in Supplementary Table S2.

**Table 2.** Cost-utility analysis of the p16/Ki67 test

Alternative	Costs	QALY	Incremental cost (R$ 2024)	Incremental effectiveness	ICER
Liquid cytology	R$11.74	0.3			
p16/Ki67	R$134.69	2.8	R$122.94	2.5	R$49.76

*Note:* QALY = quality-adjusted life years; ICER = incremental cost-effectiveness ratio.

P16/Ki67 DS detected 198 additional lesions per 1,000 women compared to cytology, including 69 cases of CIN 2+. There was also a 62 percent reduction in undue referrals for colposcopy. More details on the comparative results of lesion detection and unnecessary referrals can be found in Supplementary Table S3.

**Figure 2.**
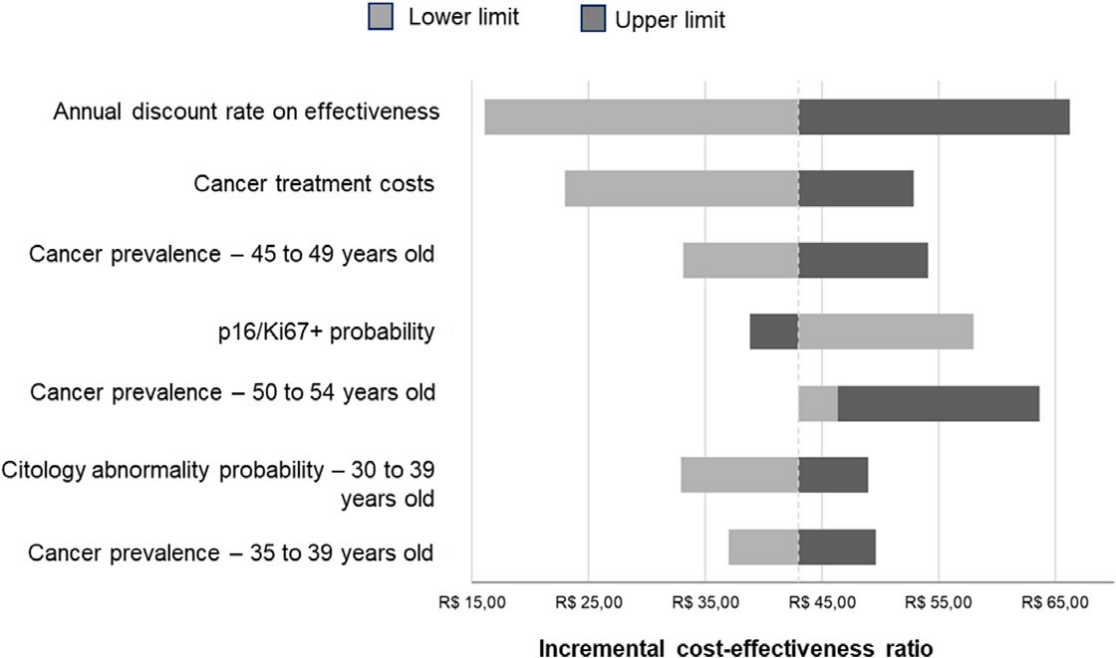
Tornado chart of the deterministic sensitivity analysis (R$ 2024).

The greatest variations in the ICER occurred with changes in the effectiveness discount rate, the cost of cancer treatment, and the prevalence of the disease between 45 and 49 years of age. Even so, DS remained cost-effective even in the worst-case scenario evaluated (Figure 2).

**Figure 3.**
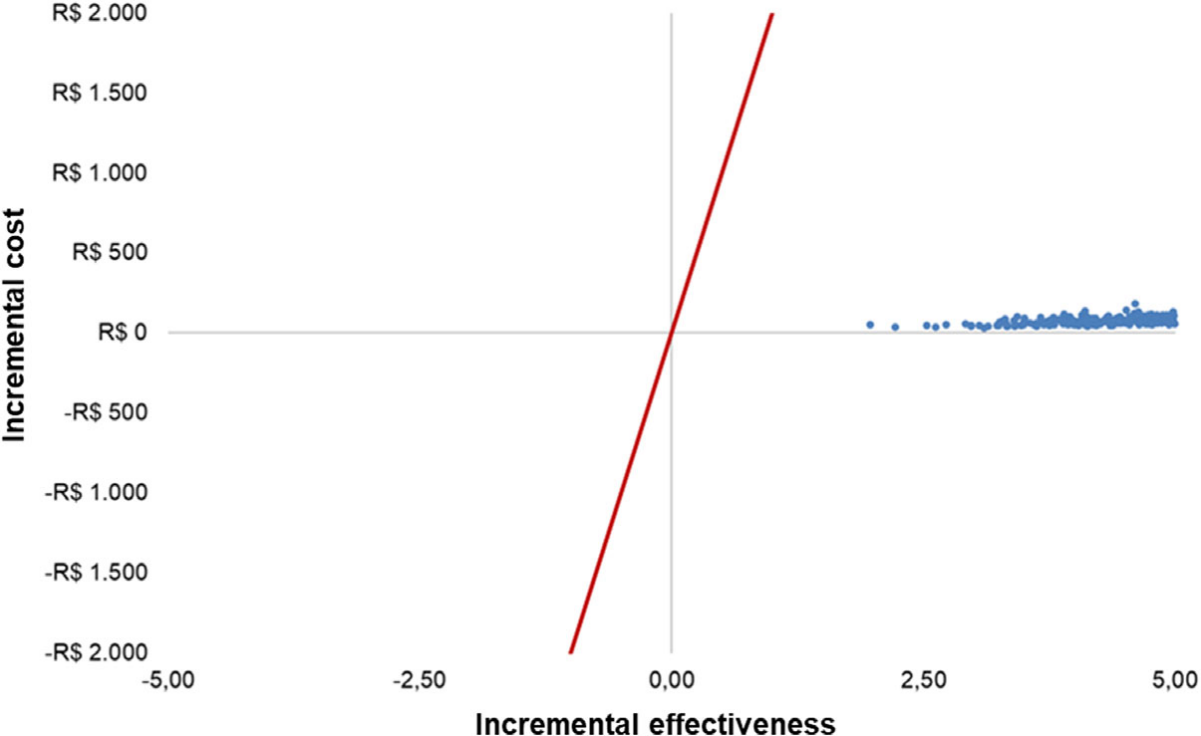
Scatter plot.

The Monte Carlo simulations were concentrated in the first quadrant of the cost-effectiveness plane, indicating that double-booking is more effective and more expensive (Figure 3). Using a graphical threshold of R$2,000, it can be seen that the ICER remained well below the cost-effectiveness threshold adopted in Brazil (R$40,000/QALY).

## Discussion

The superior accuracy of P16/Ki67 DS over LC is well established (6). In this study, we found that using DS as triage part of HPV algorithm could improve screening performance.

The gain in sensitivity without a decrease in specificity of DS, combined with the high prevalence of CIN in the modeled population, resulted in an increased number of women with precancerous lesions detected and treated early.

Consequently, these women remained for most of the time horizon in health states with higher utility and lower management costs, accumulating more QALYs throughout their lifetime without significantly increasing the associated costs. Therefore, DS was cost-effective, with an ICER well below the threshold of R$40,000/QALY.

In the deterministic sensitivity analysis, the variation in the cost of cancer treatment, the highest in the analysis, simulated scenarios of early and late diagnosis, and in both, DS remained cost-effective, even with a 1.82-fold increase in cost (variation from R$22.97 to R$52.88/QALY) compared to LC.

In five international studies identified in a previous systematic review (29), cytology was not the most cost-effective strategy in screening for ASC-US or LSIL. Cost-effectiveness studies comparing the two tests are scarce. Two studies were found that analyzed similar contexts in Thailand and Kenya, in which DS was also the most cost-effective test as screening after HPV testing (30;31). According to the literature searches carried out, this is the first economic evaluation of screening strategies in HPV+ women in Brazil. However, this study has some limitations.

A limitation is that the incremental QALY gain observed in our model may appear higher than in some international studies (30;31). This difference reflects the specific comparison performed (DS versus cytology alone, without HPV primary screening), the high prevalence of CIN in the modeled population, and the lifetime horizon adopted. In addition, the regression rates of CIN1/2 obtained from meta-analyses imply that many women return to the Normal health state after early detection and treatment, remaining in higher-utility states for most of the lifetime horizon.

To mitigate potential overestimation, we applied conservative parameter values and explored uncertainty through sensitivity analyses, which consistently confirmed the robustness and direction of the benefit.

The cost of DS was based on the purchase price of a bid from just one center, given the limitations of the official databases, such as the lack of specification of items and the number of tests carried out with the material purchased. This value was considered to be the closest to reality. This uncertainty was explored in the deterministic sensitivity analyses.

In the Brazilian guideline being updated (4), women with a negative cytology or dual-stain result are recommended to repeat the HR-HPV test after 12 months, and if positive, undergo cytology or DS again. In our model, this retesting step was not included. To minimize bias from this simplification, QALYs and costs were applied immediately after screening. Given that the average persistence of non 16/18 HR-HPV is approximately 11 months, these women would likely not re-enter the cohort (32).

Prevalences of cervical lesions from SISCAN based on histopathological results after LSIL and ASC-US were used, due to the current way of organizing the database, which follows screening with cytological examination, and because non 16/18 HR-HPV has a lower association with CIN 2+, although there are documented cases (33). The impact of prevalence on results is more related to QALYs than to costs. There may be underreporting of records, but the data follows trends compatible with the real-world scenario.

The findings of this study point to opportunities for future research with real-world data and analysis from societal perspective, including indirect costs and broader social impacts such as productivity losses, patient time, and non‑medical expenses. The evidence obtained is in line with the WHO recommendation and can support decisions on guidelines, clinical practices, and policies for cervical cancer prevention in Brazil.

## Supporting information

10.1017/S0266462326103675.sm001 Meirelles et al. supplementary material Meirelles et al. supplementary material

## Supplementary material

The supplementary material for this article can be found at http://doi.org/10.1017/S0266462326103675.
