# Supplementary material for: Cost-utility of screening with liquid cytology or p16/Ki67 dual stain in women identified in cervical cancer triage with non 16/18 HR-HPV
Source: Int J Technol Assess Health Care. 2026 Mar 30;42(1):e48. doi: 10.1017/S0266462326103675 (PMC13161926; doi:10.1017/S0266462326103675)
Supplement: Meirelles et al. supplementary material [file S0266462326103675sup001.docx]

Table 1. Probability distributions applied to cost-utility analysis parameters

| **Parameter Description** | **Distribution** |
| --- | --- |
| Average entry age – group 25–34 years | Uniform |
| Average entry age – group 35–60 years | Uniform |
| Sensitivity of liquid cytology (CIN 2+) | Beta |
| Specificity of liquid cytology (CIN 2+) | Beta |
| Sensitivity of p16/Ki67 (CIN 2+) | Beta |
| Specificity of p16/Ki67 (CIN 2+) | Beta |
| Frequency of p16/Ki67+ | Beta |
| Frequency of cytology abnormality | Beta |
| Prevalence of CIN 1 – 25–29 years | Dirichlet |
| Prevalence of CIN 1 – 30–34 years | Dirichlet |
| Prevalence of CIN 1 – 35–39 years | Dirichlet |
| Prevalence of CIN 1 – 40–44 years | Dirichlet |
| Prevalence of CIN 1 – 45–49 years | Dirichlet |
| Prevalence of CIN 1 – 50–54 years | Dirichlet |
| Prevalence of CIN 1 – 55–60 years | Dirichlet |
| Prevalence of CIN 2 – 25–29 years | Dirichlet |
| Prevalence of CIN 2 – 30–34 years | Dirichlet |
| Prevalence of CIN 2 – 35–39 years | Dirichlet |
| Prevalence of CIN 2 – 40–44 years | Dirichlet |
| Prevalence of CIN 2 – 45–49 years | Dirichlet |
| Prevalence of CIN 2 – 50–54 years | Dirichlet |
| Prevalence of CIN 2 – 55–60 years | Dirichlet |
| Prevalence of CIN 3 – 25–29 years | Dirichlet |
| Prevalence of CIN 3 – 30–34 years | Dirichlet |
| Prevalence of CIN 3 – 35–39 years | Dirichlet |
| Prevalence of CIN 3 – 40–44 years | Dirichlet |
| Prevalence of CIN 3 – 45–49 years | Dirichlet |
| Prevalence of CIN 3 – 50–54 years | Dirichlet |
| Prevalence of CIN 3 – 55–60 years | Dirichlet |
| Prevalence of cervical cancer – 25–29 years | Dirichlet |
| Prevalence of cervical cancer – 30–34 years | Dirichlet |
| Prevalence of cervical cancer – 35–39 years | Dirichlet |
| Prevalence of cervical cancer – 40–44 years | Dirichlet |
| Prevalence of cervical cancer – 45–49 years | Dirichlet |
| Prevalence of cervical cancer – 50–54 years | Dirichlet |
| Prevalence of cervical cancer – 55–60 years | Dirichlet |
| Cost of liquid cytology | Gamma |
| Cost of p16/Ki67 test | Gamma |
| Average cost of colposcopy | Gamma |
| Average cost of biopsy | Gamma |
| Average cost of excisional procedures | Gamma |
| Cost of cancer treatment | Gamma |
| Utility of cancer state | Beta |
| Annual discount rate – costs | Uniform |

*Notes: CIN = cervical intraepithelial neoplasia*

Table 2. QALYs and costs (R$ 2024) disaggregated according to the states CIN 1, CIN 2, CIN 3, Normal and Cancer for the alternatives compared

*Notes: CIN = cervical intraepithelial; QALY = quality-adjusted life years*

| Alternative | Decision tree | CIN 1 | CIN 2 | CIN 3 | Normal | Cervical cancer | Total |
| --- | --- | --- | --- | --- | --- | --- | --- |
|  | **QALY** | | | | | | |
| Liquid cytology | 6.04 | 15.81 | 3.95 | 3.95 | 307.79 | 0.14 | 337.68 |
|  | **Costs** | | | | | | |
|  | R$ 401.84 | R$ 542.87 | R$ 1,341.42 | R$ 1,088.76 | - | R$ 8,370.05 | R$ 11,744.94 |
|  | **QALY** | | | | | | |
| p16/Ki67 | 4.90 | 167.91 | 49.74 | 75.98 | 2,504.94 | 4.98 | 2,808.44 |
|  | **Costs** | | | | | | |
|  | R$ 1,943.46 | R$ 5,499.42 | R$ 11,399.67 | R$ 10,700.32 | - | R$ 105,143.85 | R$ 134,686.72 |

Table 3. Comparative triage results for cervical cancer: lesions detected, false-negatives and false-positives

|  | 25-29 yo | 30-34 yo | 35-39 yo | 40-44 yo | 45-49 yo | 50-54 yo | 55-60 yo | Total |
| --- | --- | --- | --- | --- | --- | --- | --- | --- |
| *Detected lesions* | 32 | 32 | 32 | 19 | 29 | 26 | 28 | 198 |
| CIN 1 | 19 | 19 | 19 | 12 | 20 | 20 | 21 | 131 |
| CIN 2 | 7 | 7 | 6 | 3 | 5 | 4 | 4 | 37 |
| CIN 3 | 6 | 5 | 7 | 3 | 3 | 3 | 2 | 30 |
| Cancer | 0,1 | 0,3 | 0,65 | 0,2 | 0,5 | 1 | 0,3 | 3 |
| *Losses (false negatives)* | 5 | 5 | 5 | 3 | 4 | 4 | 4 | 30 |
| CIN 1 | 3 | 3 | 3 | 2 | 2 | 4 | 3 | 20 |
| CIN 2 | 1 | 1 | 1 | 0 | 1 | 1 | 1 | 6 |
| CIN 3 | 1 | 1 | 1 | 1 | 0 | 1 | 0 | 5 |
| Cancer | 0 | 0 | 0 | 0 | 0 | 0 | 0 | 0 |
| *Unnecessary referrals*  *(false positives)* | -48 | -33 | -34 | -34 | -31 | -27 | -30 | -237 |

*Notes: yo = years old;* *CIN = cervical intraepithelial neoplasia*
